# The effect of maternal NODAL on STOX1 expression in extravillous trophoblasts is mediated by IGF1

**DOI:** 10.1371/journal.pone.0202190

**Published:** 2018-08-09

**Authors:** Allerdien Visser, Maarten Beijer, Cees B. M. Oudejans, Marie van Dijk

**Affiliations:** 1 Amsterdam UMC, VU University Amsterdam, Department of Clinical Chemistry, Amsterdam Reproduction & Development, Amsterdam, The Netherlands; 2 Amsterdam UMC, University of Amsterdam, Reproductive Biology Laboratory, Amsterdam Reproduction & Development, Amsterdam, The Netherlands; Shanghai Jiao Tong University, CHINA

## Abstract

The number of molecules identified to be involved in communication between placenta and decidua is fast expanding. Previously, we showed that NODAL expressed in maternal endometrial stromal cells is able to affect NODAL and STOX1 expression in placental extravillous trophoblasts. The effect of maternal NODAL on placental NODAL expression is achieved via Activin A, while preliminary data suggests that maternal NODAL affects STOX1 expression in trophoblasts potentially via IGF1. In the current study, T-HESC endometrial stromal cells were treated with siRNAs against *NODAL* after which *IGF1* mRNA expression was determined by quantitative RT-PCR, while IGF1 secretion was measured by ELISA. Recombinant IGF1 and inhibitors of the MAPK and PI3K/AKT pathways were added to SGHPL-5 extravillous trophoblasts after which the effects on *STOX1* mRNA and STOX1 protein expression were determined. The effect of IGF1 and the MAPK and PI3K/AKT inhibitors on the invasive capacity of SGHPL-5 cells was investigated by performing invasion assays. We found that T-HESC cells treated with *NODAL* siRNAs showed significant upregulation of *IGF1* mRNA expression and IGF1 protein secretion. Addition of IGF1 to SGHPL-5 cell media significantly upregulated *STOX1* mRNA and protein expression. Using inhibitors of the PI3K/AKT and MAPK pathway showed that the effect of IGF1 on STOX1 expression is accomplished via MAPK signaling. Secondly, PI3K inhibition independently leads to reduced STOX1 expression which can be rescued by adding IGF1. IGF1 was unable to influence the invasive capacity of SGHPL-5 cells, while inhibiting the PI3K/AKT pathway did reduce the invasion of these cells. To conclude, here we show that downregulated NODAL expression in endometrial stromal cells, previously associated with pre-eclampsia like symptoms in mice, increases IGF1 secretion. Increased levels of IGF1 lead to increased expression levels of STOX1 in extravillous trophoblasts via the MAPK pathway, hereby identifying a novel signaling cascade involved in maternal-fetal communication.

## Introduction

During early pregnancy extravillous trophoblasts invade the placental bed, modify the spiral arteries from low flow, high resistance to high flow, low resistance vessels in order to reach the demands of the developing fetus [1]. Problems in placental development can lead to early pregnancy loss and growth restriction of the fetus, but can also lead to diseases occurring in the mother such as pre-eclampsia and HELLP syndrome. Pre-eclampsia (PE), a human pregnancy-associated disease characterized by maternal hypertension and proteinuria, affects 2–8% of all pregnancies and remains a leading cause of maternal and perinatal morbidity and mortality [2]. While the clinical symptoms occur in the second half of pregnancy, the origin can be found in the first trimester. In PE patients aberrant trophoblast differentiation leads to limited invasion of extravilllous trophoblasts into the uterus followed by poor remodeling of the spiral arteries [1].

The communication between embryo and mother is one of the key processes in establishing a functional placental and decidual network. It becomes evident that besides hormones also a large number of factors like cytokines and growth factors, such as members of the transforming growth factor beta (TGF-β) superfamily, are essential [3]. The TGF-β superfamily is a large family of proteins that consists of growth and differentiation factors that are responsible for cell differentiation, proliferation and embryo development [3]. We previously studied the relation between STOX1 and TGF-β superfamily members NODAL and Activin A [4, 5]. *NODAL* is located in the same chromosomal linkage area as the Dutch PE susceptibility gene *STOX1*, a transcription factor which is associated with the familial form of early-onset, IUGR (intrauterine growth restriction)-complicated PE. Uterine nodal knockout mice presenting with an IUGR and spontaneous preterm birth phenotype [6] showed an upregulation of placental *nodal* and *stox1* mRNA expression [4]. In a human *in vitro* model system in which conditioned media from decidua tissues was added to extravillous trophoblast cells a similar effect of decidual NODAL on placental *NODAL* and *STOX1* expression was observed [4]. It was furthermore concluded that the effects observed were achieved by the undifferentiated decidual cells, i.e. endometrial stromal cells [4]. While the effect of maternal NODAL on placental *NODAL* was found to be achieved via Activin A signaling, it is not yet clear how decidual NODAL is able to affect placental STOX1 expression. However, preliminary results obtained from a human TGF-Beta/BMP signaling pathway PCR array on decidua tissues and endometrial stromal samples with downregulated NODAL expression showed upregulated levels of IGF1 mRNA (S1 Fig). As the IGF axis controls fetal growth, and reduced fetal growth is associated with abnormal placental development [7], the aim of this study was to investigate if the effect of decidual NODAL on STOX1 expression in the extravillous trophoblast is mediated by IGF1.

## Materials and methods

### T-HESC cell culture and transfection

T-HESC endometrial stromal cells were obtained from ATCC and grown in phenol-red free DMEM/F12 media supplemented with charcoal treated FBS, ITS+, pen-strep and puromycin at 37ºC, 5% CO_2_. 150,000 T-HESC cells were transfected with 6 pmol *NODAL* or negative control siRNAs (Qiagen, Germany, Flexitube Genesolution, a package of 4 preselected siRNAs for a target gene) using 1 μl lipofectamine RNAiMAX transfection reagent (ThermoFisher, USA). 48 hours after transfection, conditioned media was collected, centrifuged to remove cells and stored at -80°C. The cells were scraped in RNABee (Tel_Test, USA) followed by RNA isolation using Qiagen RNeasy mini kit.

### SGHPL-5 cell culture and incubation with IGF1 or pathway inhibitors

SGHPL-5 extravillous trophoblast cells, kindly provided by Dr Judith Cartwright, St George’s University of London, UK, were incubated with 0, 2, 20, 100 and 200 ng/ml recombinant human IGF1 protein (ThermoFisher, USA) in serum-free media. After 48h SGHPL-5 cells were collected and RNA was isolated as described above for T-HESC cells. For Western blot, protein lysates were obtained from cells treated with 200ng/ml IGF1, 50μM LY294002 (Sigma-Aldrich, USA), 50μM U0126 (Sigma-Aldrich, USA), or in combination.

### Reverse transcription quantitative real-time PCR

Quantitative RT-PCRs were performed in triplicate on an ABI7300 using the RNA-to-Ct kit (ThermoFisher, USA) with a gene expression assay for *IGF1* (Hs01547656_m1, ThermoFisher, USA) and *STOX1* (Hs03645108_m1, ThermoFisher, USA). Normalization was done with a gene expression assay for *GAPDH* (Hs00266705_g1, ThermoFisher, USA). *NODAL* siRNA-mediated knockdown was assessed with a gene expression assay for *NODAL* (Hs00415443_m1, ThermoFisher, USA). Relative mRNA expression levels were calculated using REL = 2^-ΔCt^, where ΔCt = Ct target gene–Ct GAPDH.

### ELISA

Conditioned media samples were concentrated using SpeedVac. The samples were then measured in duplicate using a commercial IGF1 ELISA kit (Sigma-Aldrich, USA) according to the manufacturer’s instructions. IGF1 levels in samples from four independent experiments performed in triplicate were measured. To compare the levels in the four different experiments IGF1 levels were normalized to the control transfection.

### Western blot

SGHPL-5 protein lysates were diluted in Laemmli buffer (Bio-Rad, USA) with β-Mercaptoethanol and heated to 95 ºC for 5 minutes. The samples were separated by SDS-polyacrylamide gel electrophoresis, and blotted onto a PVDF-membrane. STOX1 (SAB3500801, Sigma-Aldrich, USA) antibody was used in combination with horseradish peroxidase-conjugated secondary antibodies (DAKO, USA). Protein bands were detected by an enhanced-chemiluminescence assay (GE Healthcare, UK) on a ChemiDoc. Actin was used as an internal loading control. Three independent experiments were performed.

### Invasion assay

SGHPL-5 cells were serum-starved for 24 hours after which 50,000 cells were plated on 100 μl 10x diluted Matrigel (Corning, USA) coated 8.0 μm cell culture inserts in the presence of 200 ng/ml IGF1, 50 μM LY294002, 50μM U0126, or in combination. Invasion took place for 48 hours after which the membranes were fixed, covered in Vectashield with DAPI (VectorLabs, USA) and coverslipped after which the underside of the membrane was counted. Counting was performed by taking pictures of 9 random fields per membrane after which ImageJ was used to count the number of cells. Data are obtained from three independent experiments with each treatment performed in triplicate.

### Statistical analyses

Statistical analyses were performed using Graphpad Prism software using unpaired t-tests or with ANOVA followed by a Bonferroni multiple comparison test. *P* values <0.05 were considered significant. Results are presented as mean ± SEM.

## Results

### Reduced *NODAL* expression in endometrial stromal cells increases expression and secretion of IGF1

**Fig 1:**
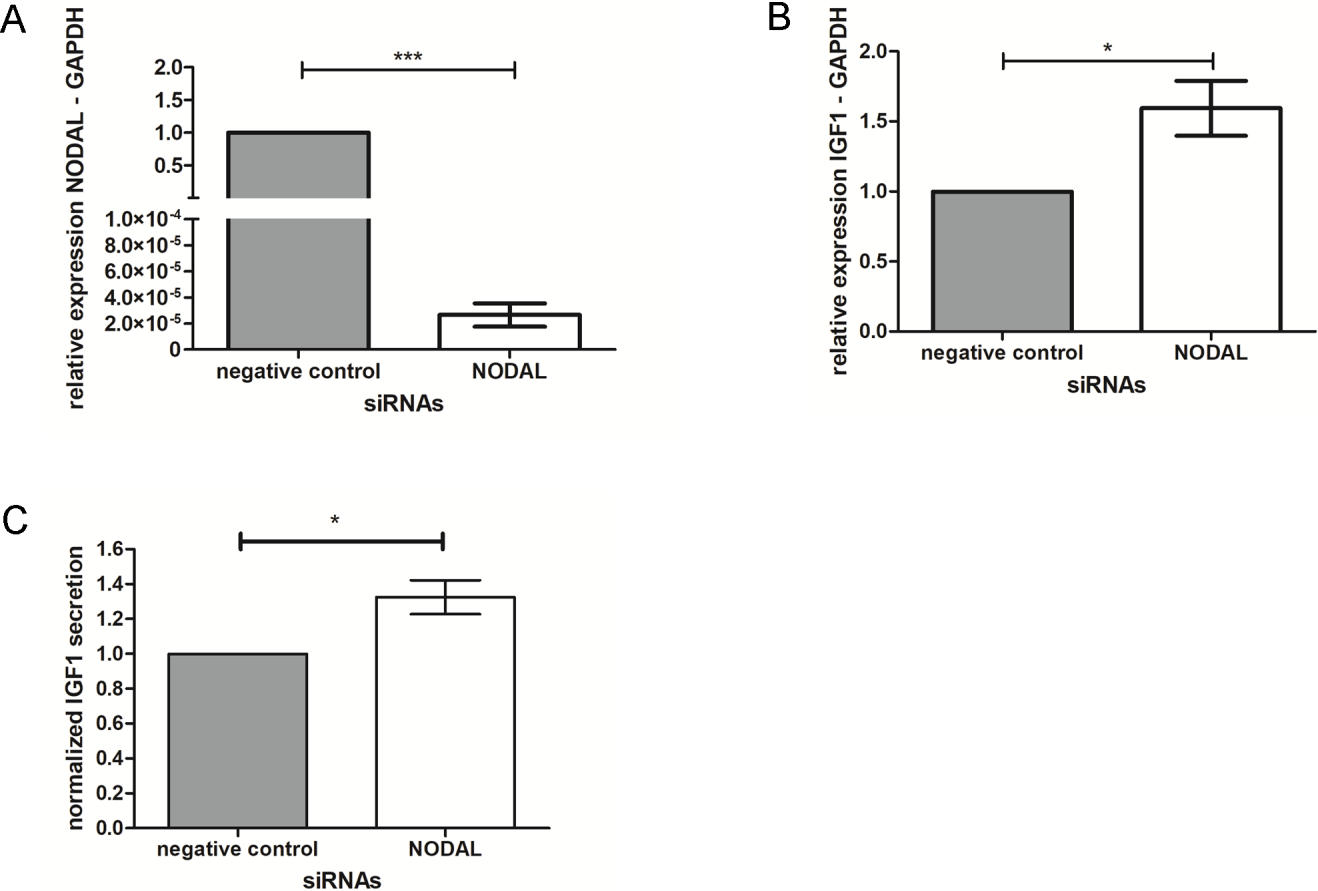
IGF1 expression and secretion upon *NODAL* knockdown. RT-qPCR measuring *NODAL* mRNA expression in T-HESC cells after transfection with *NODAL* or negative control siRNAs confirming *NODAL* knockdown was successful **(A)**. RT-qPCR measuring *IGF1* mRNA expression in T-HESC cells after transfection with *NODAL* or negative control siRNAs showing upregulation of *IGF1* upon *NODAL* knockdown **(B)**. ELISA measurements show an increase in secreted IGF1 levels in T-HESC cell media upon NODAL silencing **(C).** Bars are mean ± SEM, * indicates P < 0.05, ** indicates P < 0.01.

Preliminary results obtained from a human TGF-Beta/BMP signaling pathway PCR array on RNA obtained from decidua tissues and the endometrial stromal cell line T-HESC [4], indicated that upon *NODAL* knockdown *IGF1* expression increased. To confirm previous findings, T-HESC cells were treated with siRNAs against *NODAL* (Fig 1A) which led to an upregulation of *IGF1* mRNA expression (Fig 1B). Secondly, in the media of these cells IGF1 protein secretion was measured by ELISA, showing an increase in IGF1 secretion upon *NODAL* knockdown (Fig 1C).

### IGF1 increases *STOX1* expression in extravillous trophoblasts

**Fig 2:**
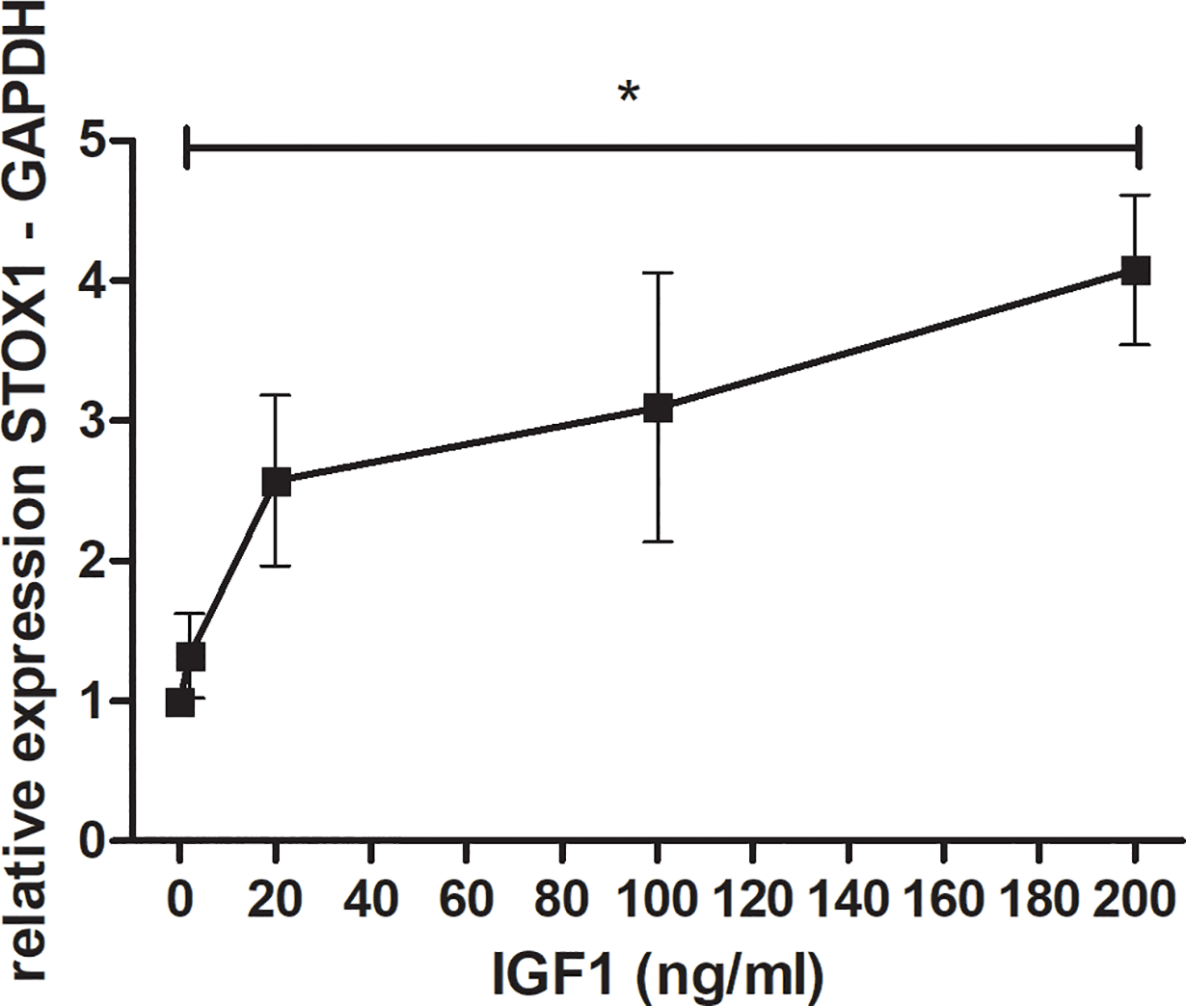
IGF1 shows a dose-dependent effect on *STOX1* mRNA levels. RT-qPCR measuring *STOX1* mRNA expression in SGHPL-5 cells after addition of 0-200ng/ml recombinant human IGF1 protein showing an increase in *STOX1* mRNA expression upon increasing levels of IGF1. Bars are mean ± SEM, * indicates P < 0.05 between 2 and 200 ng/ml IGF1.

To investigate if IGF1 has a direct influence on *STOX1* expression in the placenta, SGHPL-5 cells, representing first trimester extravillous trophoblasts, were incubated with different concentrations of recombinant IGF1 where 2 ng/ml was considered physiologic (ED_50_), while 100–200 ng/ml was considered to be a pathologic concentration in cell culture based on the increase found in circulating IGF1 in normal and pre-eclamptic patients [8]. *STOX1* mRNA expression was found to increase with increasing levels of IGF1 (Fig 2).

### IGF1 increases STOX1 protein levels via the MAPK pathway

**Fig 3:**
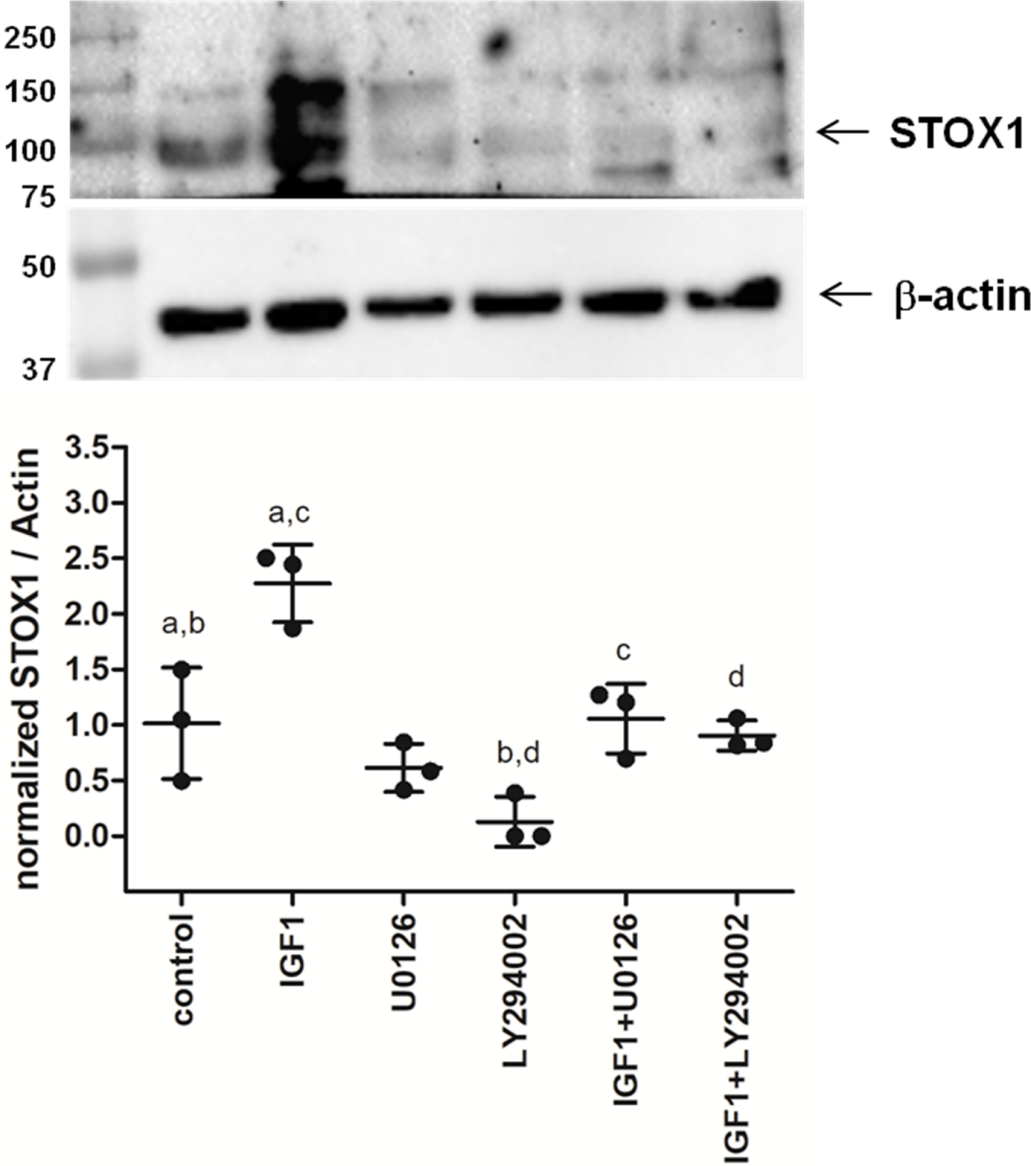
Increased STOX1 protein biosynthesis upon IGF1 treatment. Representative Western blot and densitometric analysis based on three independent experiments of STOX1 protein in SGHPL-5 cells treated with IGF1, PI3K inhibitor LY294002 or MAPK inhibitor U0126 alone or in combination. IGF1 significantly increases STOX1 protein synthesis. LY293002 significantly reduces STOX1 protein levels which can be rescued by IGF1. U0126 does not affect STOX1 expression but does reverse the effect of IGF1 treatment. Bars are mean ± SEM. a, c indicate P < 0.01; b, d indicate P < 0.05.

IGF1 in the placenta signals through the PI3K/AKT and the MAPK pathway promoting proliferation, differentiation and invasion [7]. As it was previously shown that STOX1 can be phosphorylated by the PI3K/AKT pathway leading to cytosolic translocation and subsequent degradation of this transcription factor [9], we decided to study the effect of IGF1 on STOX1 protein expression in SGHPL-5 cells. After incubation with 200 ng/ml IGF1 the amount of STOX1 present in the cells significantly increased compared to the control situation (Fig 3). Furthermore, when inhibiting PI3K by adding LY294002, STOX1 levels significantly decreased as also observed before [9]. This decrease could be rescued by adding IGF1. Addition of the MAPK inhibitor U0126 did not affect STOX1 protein expression on its own, however, U0126 was able to reverse the effect seen upon IGF1 addition. These results indicate that the effect of IGF1 on STOX1 expression is occurring via the MAPK signaling pathway.

### IGF1 does not influence invasion

**Fig 4:**
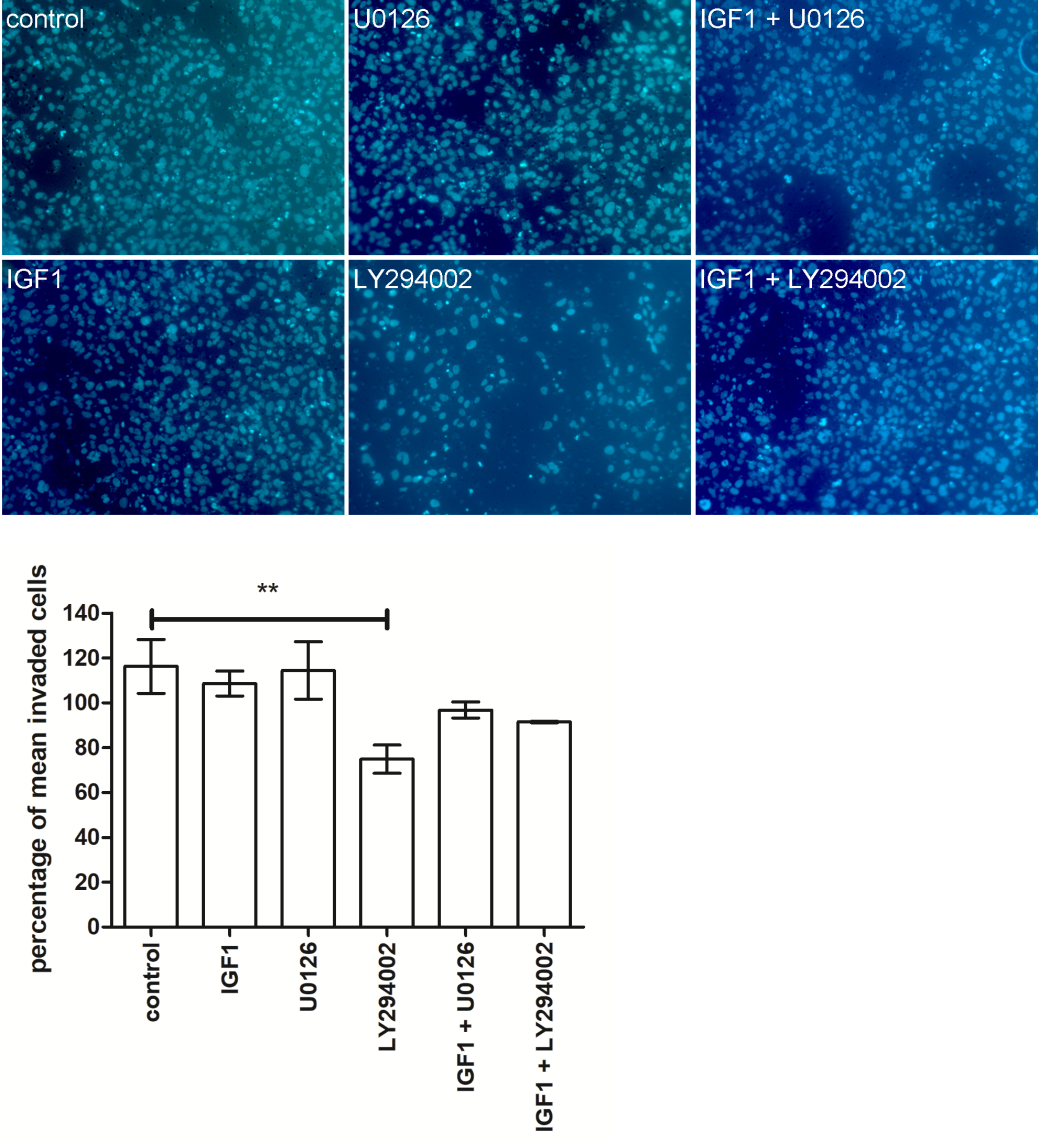
IGF1 has no effect on SGHPL-5 cell invasiveness. Representative images and graph of transwell invasion assays using SGHPL-5 cells treated with IGF1, PI3K inhibitor LY294002 or MAPK inhibitor U0126 alone or in combination. IGF1 has no effect on invasion. Inhibiting PI3K by adding LY294002 decreases invasion of SGHPL-5 cells significantly. Bars are mean ± SEM, ** indicates P < 0.01.

One of the functional endpoints associated with induced STOX1 expression is a decrease in the invasive capacity of SGHPL-5 cells [9]. To investigate if IGF1 signaling is also involved in this pathway transwell invasion assays were performed. These showed no effect of IGF1 on the invasion of SGHPL-5 cells (Fig 4). Also no effect of inhibition of MAPK by addition of U0126 was observed. A small but significant effect of PI3K inhibitor LY294002 was seen as the amount of invaded SGHPL-5 cells decreased upon addition of this inhibitor.

## Discussion

This study was designed to investigate how decidual NODAL is able to affect placental STOX1 expression. Here, we describe that downregulating NODAL in endometrial stromal cells increases IGF1 secretion which in turn leads to increased expression levels of STOX1 in extravillous trophoblasts through the MAPK signaling pathway.

Firstly, we show that silencing of *NODAL* in endometrial stromal cells leads to an increase in the expression and secretion of IGF1. It is known that uterine knockdown of Nodal in mice leads to a pre-eclampsia-like phenotype [6]. It is still unclear if maternal IGF1 in early pregnancy is associated with the development of pre-eclampsia, but it is thought to play a role in placentation and is therefore well studied [10]. The majority of studies performed on circulating IGF1 levels in the mother find a decrease in pre-eclamptic pregnancies [11]. However, a few studies find the opposite [12, 13], and a case-control study performed in Norwegian women describes an increase in serum IGF1 levels from first to second trimester as a predictor for preterm pre-eclampsia [8].

Secondly, we show that following addition of increasing levels of IGF1 to the media of the extravillous trophoblast cell line SGHPL-5, these cells increase their *STOX1* mRNA expression levels in a dose-dependent manner. Furthermore, we show that high IGF1 levels cause an increase in biosynthesis of STOX1 protein in these cells. STOX1 is known to function as a transcription factor regulating genes that increase trophoblast proliferation and decrease trophoblast invasion [9, 14, 15]. In the placenta IGF1 binding to its receptor results in the activation of the PI3K/AKT pathway and MAPK pathway, both resulting in transcription of genes involved in proliferation, differentiation and invasion [7]. We previously showed that STOX1 can be phosphorylated by AKT leading to an increase in cytoplasmic localization and degradation [9]. This knowledge would suggest that increased IGF1 levels would decrease STOX1 levels. As we found an upregulation of STOX1 upon increased IGF1 levels we decided to investigate this unexpected effect further by using inhibitors of PI3K and MAPK. By using these inhibitors we show that the effect of IGF1 on STOX1 expression is accomplished via MAPK signaling, where IGF1 leads to an increase in STOX1 mRNA transcription and subsequent protein synthesis. As shown before [9], PI3K inhibition independently leads to reduced (cytoplasmic) STOX1 levels, which in turn can be rescued by adding IGF1.

Reduced invasion is one of the functional endpoints associated with induced STOX1 expression [9], however, we did not observe an effect of IGF1 on the invasive capacity of SGHPL-5 cells. This might reflect the possibility that the effects of STOX1 on invasion are accomplished via PI3K/AKT signaling only [9], or that the effects are masked by induced proliferation of the invading cells. Previous studies also found limited effects of IGF1 on invasion in first trimester extravillous trophoblast cultures [16, 17], while an effect of IGF1 on proliferation seems to be observed more consistently in both extravillous trophoblast cell and placental explant cultures [17–20]. As STOX1 is also associated with a positive effect on proliferation [21], this would be the proposed downstream effect IGF1 achieves via STOX1. However, although both IGF1 and STOX1 are known to be involved in increasing proliferation, they also both accomplish this by signaling via multiple pathways. Further investigating this proposed effect will therefore not yield definitive answers, similarly as observed in the invasion assays.

A small effect of PI3K inhibitor LY294002 on invasion was seen as the number of invaded SGHPL-5 cells decreased. However, as the PI3K/AKT pathway is a commonly used signaling pathway this effect most likely is not caused by the observed decrease in STOX1 levels upon addition of this inhibitor specifically, but rather caused by reduced transcription of several cell survival genes.

Future research should focus on further investigating this and other pathways involved in maternal-fetal communication in a model more closely resembling the human in vivo situation. This could consist of a co-culture of decidual cells and trophoblasts or, preferably, a co-culture of first trimester decidual and placental explants [22, 23].

To summarize, here we show that downregulated NODAL expression in uterine endometrial stromal cells, previously associated with pre-eclampsia like symptoms in mice, increases IGF1 secretion. Increased levels of IGF1 lead to increased expression levels of STOX1 in extravillous trophoblasts via the MAPK pathway, hereby identifying a novel signaling cascade involved in maternal-fetal communication potentially leading to changes in proliferation and subsequent differentiation of extravillous trophoblasts.

## Supporting information

S1 Fig Human TGF-Beta/BMP signaling pathway PCR array on decidua tissue and endometrial stromal samples with downregulated NODAL expression. (PDF)
