# Supplementary material for: The effect of maternal NODAL on STOX1 expression in extravillous trophoblasts is mediated by IGF1
Source: PLoS One. 2018 Aug 9;13(8):e0202190. doi: 10.1371/journal.pone.0202190 (PMC6084977; doi:10.1371/journal.pone.0202190)
Supplement: S1 Fig — (PDF) [file pone.0202190.s001.pdf]

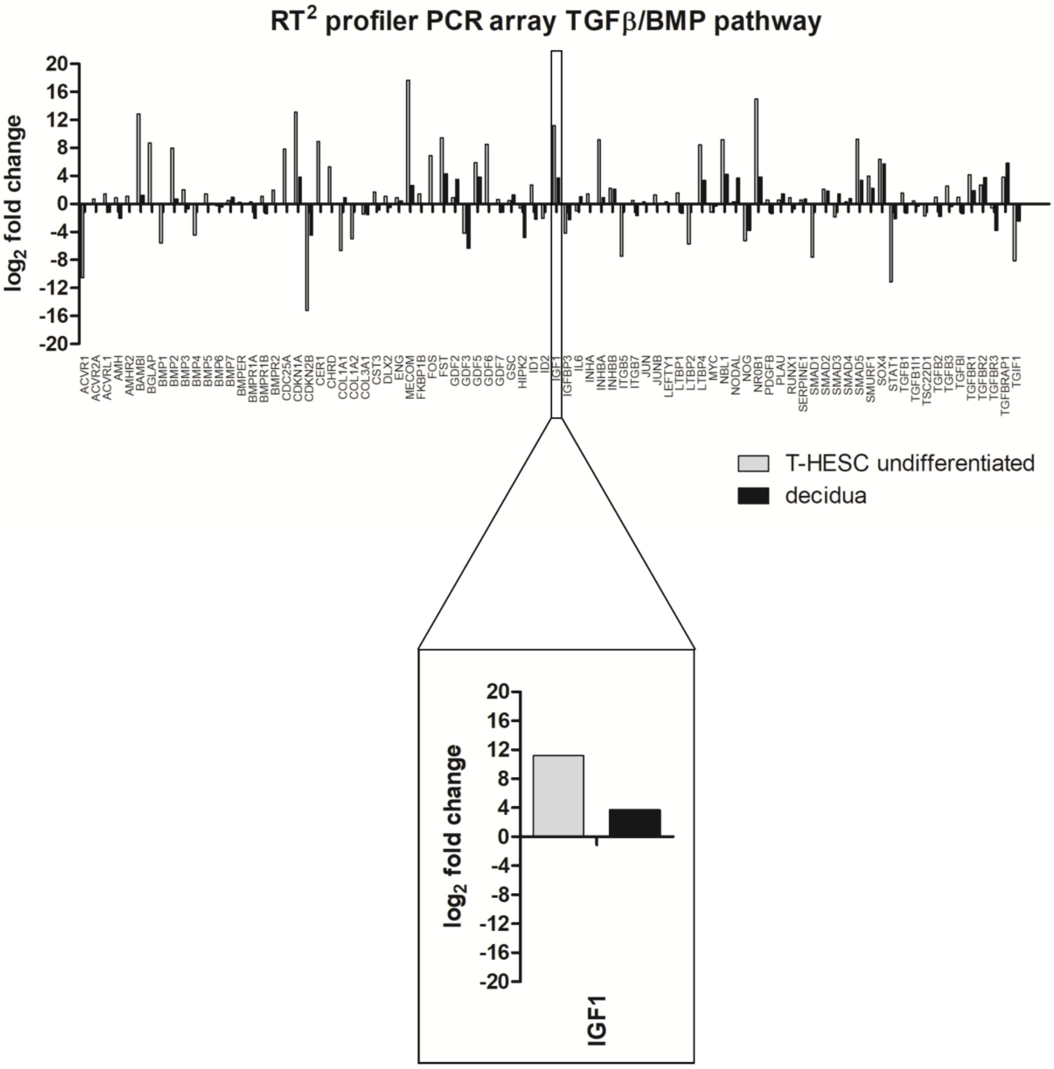

S1 Figure. Human TGF-Beta/BMP signaling pathway PCR array on decidua tissue and endometrial stromal samples with downregulated NODAL expression. Log_2 fold change of TGF-Beta/BMP signaling pathway genes in decidua tissue and undifferentiated T-HESC cells transfected with NODAL siRNAs relative to tissue or cells transfected with scrambled control siRNAs.
